# Amino acid assisted synthesis of CDs: a novel paradigm in plant tissue culture media for enhanced cellular effects and biotechnological advancements†

**DOI:** 10.1039/d4ra08776c

**Published:** 2025-01-28

**Authors:** C. Annmary, Bitty Joseph, N. J. Simi, V. V. Ison

**Affiliations:** a Centre for Nano Bio Polymer Science and Technology, Department of Physics, St. Thomas College Palai Kerala 686574 India isonv@rediffmail.com +919446126926; b Department of Physics, St. George's College Aruvithura Kerala 686122 India; c Department of Physics, Newman College Thodupuzha Kerala 685585 India; d Department of Physics, Kuriakose Elias College Mannanam Kerala 686561 India

## Abstract

We report a green approach to prepare carbon dots (CDs) with fresh tomatoes as carbon sources and amino acids as dopants (ACDs) by a microwave assisted method. The synthesised CDs were analysed by UV-visible absorption spectroscopy, photoluminescence spectroscopy, high resolution transmission electron spectroscopy, X-ray diffraction, Fourier transform infrared spectroscopy, X-ray photo electron spectroscopy. An MTT assay was used to evaluate the cytotoxicity of CDs toward L929 cells and found that CDs exhibit low cytotoxicity. The effects of ACDs on seed germination were evaluated by treating pea seeds with various concentrations of CDs. The results demonstrated a significant increase in germination and seedling vigour compared to untreated controls. Subsequently, the application of ACDs was extended to plant tissue culture. Explants treated with ACDs exhibited enhanced growth and development, indicating improved morphogenesis and proliferation rates. This study highlights the potential of ACDs as an efficient, non-toxic growth promoter in both seed germination and plant tissue culture, paving the way for their application in sustainable agriculture and plant biotechnology.

## Abstract 2

Green synthesis of fluorescent carbon dots with fresh tomatoes as carbon sources and amino acids as dopants (ACDs) by a microwave assisted method for enhanced seed germination and tissue culture applications.

## Graphical abstract

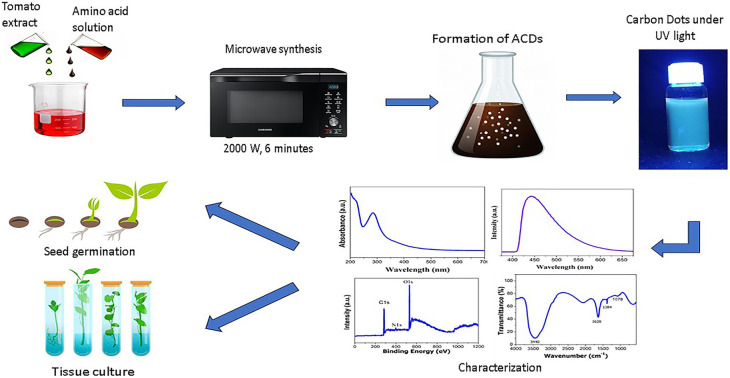

## 1. Introduction

In nanotechnology, the search for novel materials has given rise to quantum dots, which are highly regarded for their distinct optical and electrical characteristics.^1,2^ Concerns regarding environmental impacts and toxicity have prompted scientists to explore alternative methodologies. The development of carbon dots (CDs)—nanomaterials with similar functions but greater sustainability—has been accelerated by this quest.^3^ CDs, derived from the idea of quantum dots, have attracted interest due to their unique characteristics and range of potential uses.

The exceptional characteristics of CDs pave the way for a wide range of potential applications. Their remarkable water solubility, strong fluorescent emission, photostability, biocompatibility, and low toxicity make them highly significant across diverse fields.^4,5^ These applications encompass a variety of fields, including biosensing, tissue imaging, sensing, drug delivery, antimicrobial and antioxidant agents, photo catalysis, fluorescent nano-thermometers, optoelectronic devices, anti-inflammatory bioactivity, detection of heavy metals, food safety, food storage, theranostics and supercapacitors.^6–18^

The application of CDs in agriculture is receiving significant interest due to their low toxicity, biocompatibility, facile synthetic strategies with easy scale-up, good solubility and stability during usage and easy tunability of surface functionality.^19^ Functionalized CDs can serve as slow-release fertilizers, promoting plant growth, as demonstrated by Wang *et al.*^20^ and as efficient nanosensors for detecting soil contaminants.^21^ A similar study of CDs, functionalized with specific biomolecules by Kang's group, identified their significant potential in plant disease diagnostics by enabling the detection of pathogens with high sensitivity and specificity.^22^ Again, they exhibit capability in managing abiotic stresses and enhancing crop yield as shown in a study by Cramer *et al.*^23^ Integrated into smart nanomaterials, CDs enable real-time monitoring of soil conditions for precision agriculture.^24^ Jing *et al.* outlined that CDs enhance photosynthesis in plants by increasing chlorophyll content, thereby improving light absorption, utilization and energy conversion efficiency within chloroplasts, ultimately optimizing the photosynthetic process.^25^ These applications underscore the significant role CDs can play in advancing agricultural practices.

Seed germination and tissue culture are fundamental techniques used in agriculture and biotechnology. Enhancing the efficiency of these processes can overcome challenges such as limited propagation rates, poor germination under stress conditions and slow tissue differentiation. Previous studies suggest that CDs can improve nutrient uptake, regulate reactive oxygen species (ROS) and facilitate electron transfer within plant cells, thus accelerating growth and improving plant health.^26^ The mechanisms through which CDs influence these processes, especially at the cellular and molecular levels, remain inadequately explored. This study aims to bridge this gap by investigating the role of CDs in seed germination and tissue culture, focusing on their ability to enhance growth rates.

We have investigated the nitrogen doping of CDs using amino acids for agriculture applications. While most of the reports on nitrogen doping utilize ammonium compounds, we explored amino acid prepared from lemon peels as the nitrogen source based on Brodkorb's methodology,^27^ a green technique. The CDs were synthesized using microwave assisted synthesis approach, a cost-effective and environmentally friendly approach. It has been found that the amino acid doped CDs (ACDs) play a significant role in seed germination, particularly in the shoot and root length. To proceed further, we have also investigated the effect of ACDs in the tissue culture media of *Oncidium brassia* explants. Understanding the impact of ACDs on tissue culture systems offers insights into optimizing conditions for plant propagation and fostering advancements in horticultural practices. To the best of our knowledge, the use of ACDs is new in the field of plant tissue culture.

## 2. Experimental section

### 2.1 Materials and methods

Fresh tomatoes and lemon were purchased from local market. Hydrochloric acid (HCl) purchased from Sigma-Aldrich. The chemicals were used as procured without further purifications. Distilled water was used as the solvent and for washing purposes.

### 2.2 Synthesis of ACDs

Brodkorb's methodology was adapted in a modified form to prepare ACDs. *Citrous limon* were obtained from local market. Peels were separated from fruits and they were dried in the shade. The dried peels were powdered with a kitchen grinder and stored for further analysis. Citrus peel powder (10 g) was added to 100 mL of 6 M hydrochloric acid, mixed carefully and incubated at 100 °C for 24 hours. The obtained black coloured ash mixed with distilled water and kept at 4 °C for doping purpose. For the synthesis of CDs doped with amino acids, the *Solanum lycopersicum* was mashed using a domestic blender to obtain homogenous mass, which was then supplemented with 4 mL of the amino acid solution prepared as described above. The solution was diluted to a total volume of 80 mL using distilled water and subsequently irradiated with 2000 W power for 6 minutes in a microwave oven. The resultant mixture (ACDs), was mixed with distilled water and made to 40 mL. The obtained ACDs were collected by removing larger particles through centrifugation, filtered with laboratory filter paper and then with a PTFE syringe filter of size 0.2 μm.

UV-vis Absorption spectra were acquired from Shimadzu UV-3600 Plus UV-visible spectrophotometer in the range 200 to 800 nm. The PL spectra were collected from a Shimadzu RF-6000 Spectro fluorophotometer at an excitation wavelength of 280 nm. FTIR analysis was performed on Perkin Elmer spectrometric analyzer in the range from 400 to 4000 cm^−1^ at room temperature. The XRD studies were carried out using Bruker AXS D8 Advance X-ray diffractometer employing Cu Kα radiation. The high-resolution transmission electron images of the CDs were recorded using JEOL JEM-2100 microscope, after depositing them on carbon coated copper grids. The elemental composition was analysed with Xray Photoelectron Spectroscopy (XPS) done using PHI 5000 Versa Probe scanning ESCA microprobe. Cytotoxicity of the CDs was assessed using standard MTT assay on L929 cells.

## 3. Results and discussion

### 3.1 Optical properties of ACDs

**Fig. 1:**
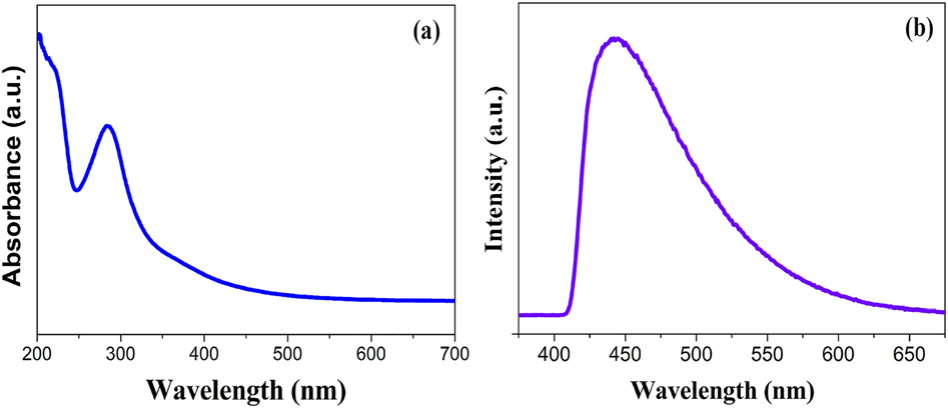
(a) UV absorption and (b) PL spectrum of ACDs.

The fluorescence emission is one of the most charming features of CDs, which has been utilized in many fields. To gain insight into the optical properties of the fabricated ACDs, UV-vis and PL studies were performed. The absorption spectrum of ACD showed distinct peaks at 235 nm and 284.8 nm as in Fig. 1a. The strong absorption band at 284.8 nm can be assigned to the n–π* transitions of the C=O bonds and the weak absorption peak centred at approximately 235 nm can be attributed to π–π* transition of the conjugated C=C bonds, which reflects the presence of conjugated π-electron systems within the CD core.^28^Fig. 1b shows the PL spectra, where ACDs exhibited excitation and emission wavelengths at 340 nm and 443.8 nm. The prepared ACD solution when kept under UV light (365 nm) exhibited green colour, the same in daylight was in a brownish yellow colour, as shown in Fig. S1.† The excitation-dependent emission behaviour of the ACDs were seen, which is one of the distinct characteristics of CDs.

### 3.2 Structural properties of ACDs

**Fig. 2:**
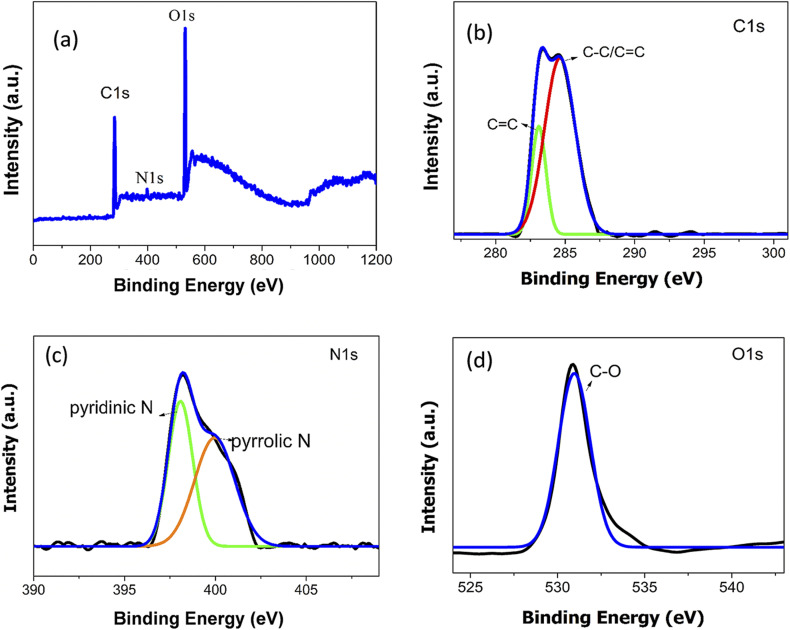
High-resolution XPS spectrum of (a) ACDs over the entire scan range (b) C1s (c) N1s and (d) O1s.

The elemental composition of synthesised ACDs were analysed using XPS and FTIR spectroscopy. The XPS spectrum Fig. 2 shows three peaks at 284.5 eV, 398.5 eV, and 531.5 eV are attributed to C1s, O1s and N1s respectively. These results indicate the presence of 54.37% of carbon, 42.96% of oxygen and 2.67% of nitrogen in the ACDs. Following the baseline correction, the peaks were deconvoluted using Gaussian–Lorentzian line shapes. The detailed spectrum of C1s spectrum shows two peaks at 283.4 eV and 284.6 eV attributed to C=C and C–C/C=C groups. The O1s has one peak at 530.9 eV assigned to C–O groups. The peaks at 398.2 eV and 400.2 eV in N1s spectrum come from pyridinic and pyrrolic Nitrogen.^29–31^ Therefore, the XPS data reveals that the surfaces of ACDs were enriched with functional groups containing carbon, nitrogen and oxygen atoms.

**Fig. 3:**
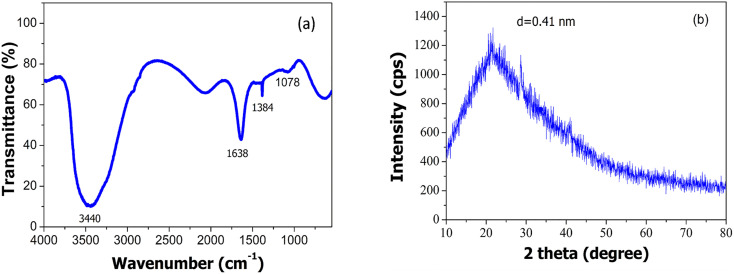
(a) FTIR spectrum and (b) XRD pattern of ACDs.

**Fig. 4:**
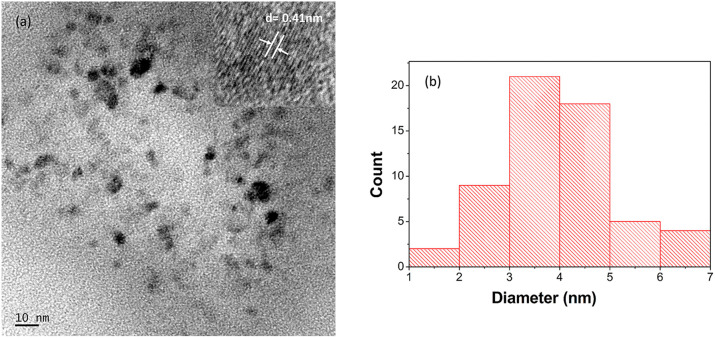
(a) TEM image of ACDs and (b) its particle size distribution.

The functional groups existing in the ACDs were characterised by FTIR spectroscopy (Fig. 3a). For ACDs, the broad absorption band at 1638 cm^−1^, 1384 cm^−1^ and 1078 cm^−1^ were ascribed to C=O, COO/C–NH and C–O–C stretching vibrations.^31–33^ The presence of carbonyl and hydroxyl bonds in ACDs impart hydrophilicity and consequently enhances their water dispersibility. In the X-ray diffraction pattern depicted in Fig. 3b, ACDs exhibit a broad diffraction peak centred at 21.6°. The corresponding interlayer distance (d) for this peak was determined to be 0.41 nm. The interlayer distance of the CDs matches with the HRTEM result and is higher than that of the graphitic carbon (0.34 nm). This confirms poor crystalline nature of CDs due to the presence of surface functionalities.^34^ The TEM images (Fig. 4a) clearly reveal that the CDs were spherical in shape with a narrow size distribution ranging between 1 nm to 7 nm (Fig. 4b).

### 3.3 Cytotoxicity

The cytotoxicity of the ACDs was assessed using standard MTT assay on L929 cells. The cells were seeded on 96 well plates at a density of (2500 cells per well) and then incubated for 24 hours under 5% CO_2_ at 37 °C. ACD samples diluted in DMEM media with varying concentrations (6.25, 12.5, 25, 50, 100 μg mL^−1^) were added to the wells containing cultured cells and further incubated for 24 hours.

**Fig. 5:**
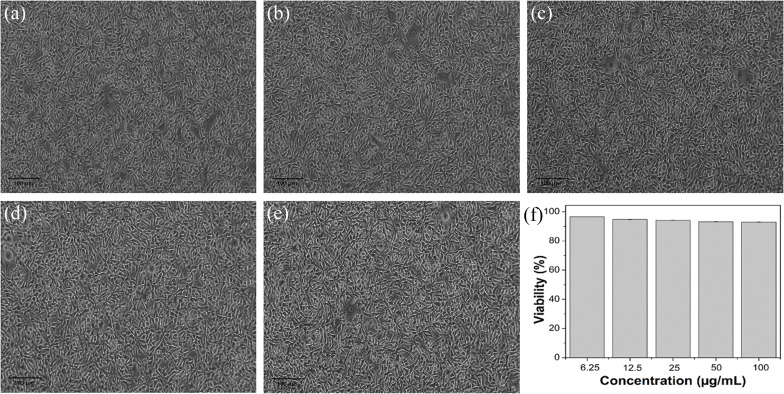
Microscopic images of L929 cells after incubation of 24 hours with various concentrations of ACDs. (a) 6.25 μg mL^−1^, (b) 12.5 μg mL^−1^, (c) 25 μg mL^−1^, (d) 50 μg mL^−1^, (e) 100 μg mL^−1^ (f) cell viability (%) of L929 cells after incubation of 24 hours with various concentrations of ACDs.

Untreated wells were kept as control. All the experiments were done in triplicate and average values were taken in order to minimize errors. The medium was removed and 100 μL of 0.5 mg mL^−1^ MTT solution in PBS were added to the wells and incubated again for 2 hours for the development of formazan crystals. The supernatant was removed and 100 μL DMSO (100%) were added per well. The absorbance at 570 nm was measured with micro plate reader.^35,36^ The cell viability was calculated using the formula:
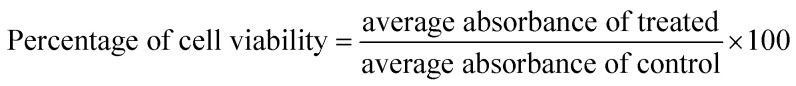


Fig. 5 shows the microscopic images of L-929 cells after incubation of 24 hours with various concentrations of ACDs. It was found that the synthesized ACDs did not affect the L-929 normal cells. Cell viability of L-929 after incubation of 24 hours with various concentrations of synthesized ACDs were greater than 90%, indicating that the sample was not cytotoxic to the normal cells.

### 3.4 Seed germination

**Fig. 6:**
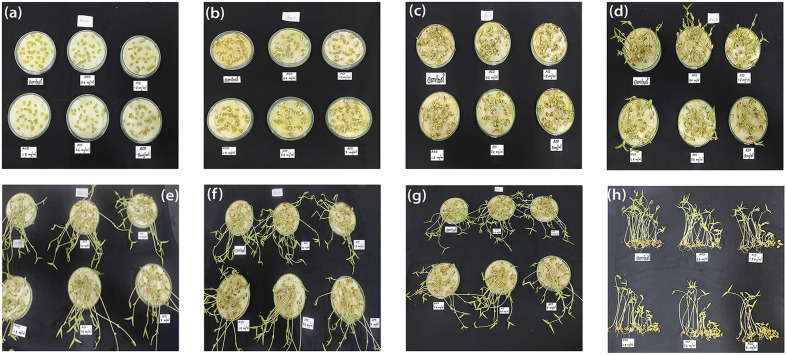
Digital photographs showing the daily response of ACDs (a–g): day 1 to day 7 and (h): vertical view of pea plant seed germination on the 7th day.

Pea seeds were immersed in a 5% sodium hypochlorite solution for 10 minutes to ensure surface sterilization and was washed four times to remove additional sterilizing solutions on surface. The specimens underwent a two-hour immersion in distilled water followed by successive soaking in suspensions of varying concentrations of ACD for an additional two hours each. In each Petri dish (100 mm × 15 mm), 30 seeds at 1 cm apart were transferred onto the filter paper and test solution was added. Petri dishes were covered and sealed with tape and kept under the laboratory condition (room temperature 27 °C) with the diffused sunlight during the daytime for 7 days. After one week, the number of germinated seeds were counted and the root and shoot length were measured.^37^Fig. 6 illustrates the daily response of pea plants from day 1 to 7, with Fig. 6h depicting the final day's response from a vertical perspective.

Germination parameters tested were as follows:

(1) Time to start germination: after 24 hours of incubation.

(2) Germination% = (germinated seeds/total seeds × 100).

**Fig. 7:**
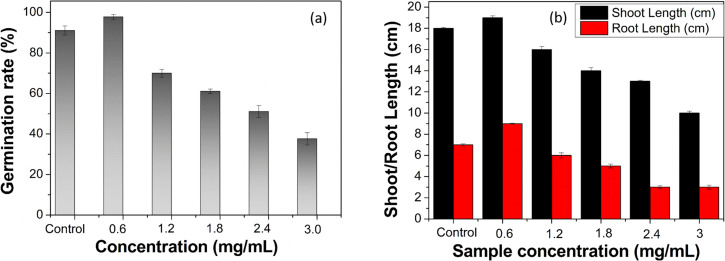
(a) Percentage of germination rate of pea plants with varying concentrations of ACDs and (b) shoot & root length of germinated pea plants with varying concentrations of ACDs.

Seed germination of pea seeds was studied in varying concentrations of ACDs. Fig. 7a shows the percentage of germination rate of pea seeds, while Fig. 7b depicts the shoot and root lengths of germinated pea plants at varying concentrations of ACDs. At a concentration of 0.6 mg mL^−1^ of ACD, the growth percentage peaks at 98%, coinciding with maximum shoot and root lengths. The small size and high surface area of CDs enable them to interact closely with plant roots, enhancing the absorption of water and essential nutrients. This improved nutrient uptake supports better growth and development.^26^ As the concentration increases beyond 0.6 mg mL^−1^, there is a consistent decline in growth percentage, shoot length and root length. This consistent pattern suggests that lower concentrations of ACD yielded superior results in plant growth. The concentration of 0.6 mg mL^−1^ of ACD appears to be the most effective for promoting the germination of pea seeds and subsequent seedling growth. Low concentrations of CDs promote plant growth, whereas higher concentrations inhibit it due to the overproduction of reactive oxygen species within plant cells and the resulting phytotoxic effects.

### 3.5 Micropropagation/tissue culture

**Fig. 8:**
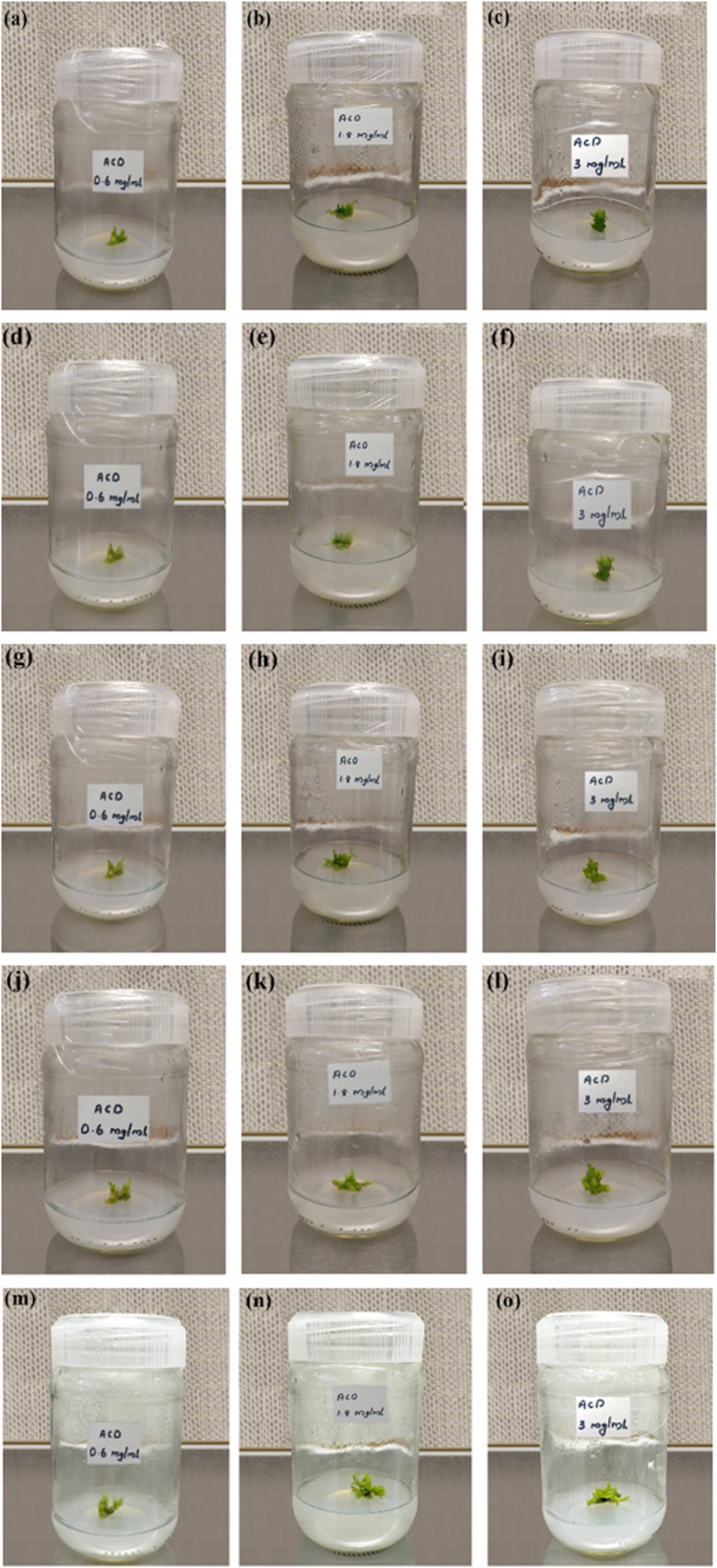
Photographs documenting the evolution of callus growth over multiple weeks showcasing the effects of different concentrations. (a–c) First week response of callus growth at a concentration of 0.6 mg mL^−1^, 1.8 mg mL^−1^ and 3 mg mL^−1^ of ACDs (d–f) second week response of callus growth at a concentration of 0.6 mg mL^−1^, 1.8 mg mL^−1^ and 3 mg mL^−1^ of ACDs (g–i) Third week response of callus growth at a concentration of 0.6 mg mL^−1^, 1.8 mg mL^−1^ and 3 mg mL^−1^ of ACDs (j–l) Fourth week response of callus growth at a concentration of 0.6 mg mL^−1^, 1.8 mg mL^−1^ and 3 mg mL^−1^ of ACDs (m–o) fifth week response of callus growth at a concentration of 0.6 mg mL^−1^, 1.8 mg mL^−1^ and 3 mg mL^−1^.

Healthy plants of *Oncidium brassia* were raised in pots containing soil under greenhouse conditions. The explants taken from these plants were used for micro propagation experiments. Collection of explants for micropropagation should be done after appropriate pre-treatment of the mother plants with fungicides and pesticides to minimize contamination in the *in vitro* cultures. This improves growth and multiplication rates of *in vitro* cultures. The explants (seeds) were washed thoroughly with water (5–7 minutes) and treated with the detergent Tween-20 for 10 minutes. The explants were then rinsed thrice with distilled water to remove excess detergent. To avoid bacterial and fungal contamination, it was further treated with bavistin (100 mg/100 mL) for 15–20 minutes and then with 2% sodium hypochloride solution for 7–8 minutes under continuous stirring, immersed in 0.1% (w/v) mercuric chloride (HgCl_2_) for few minutes for surface sterilization and then washed repeatedly with distilled water thrice to remove the disinfectants properly. The cut ends of the explants were again trimmed with the help of sterile blade to eliminate any possible residue of surfactant and the explants were used for culturing. ACDs of different concentrations 0.6 mg mL^−1^, 1.8 mg mL^−1^ and 3 mg mL^−1^ were taken for analysis. Callus developed from seeds of *Oncidium brassia* was used for this particular study. The pH of tissue culture media was adjusted between 5 to 7 before gelling and sterilization with the help of dilute NaOH.^38^ Images of week responses (Fig. 8) show the cellular growth in tissue culture media. Understanding the impact of ACDs on cell proliferation can provide insights into its use as a growth-promoting agent.

**Fig. 9:**
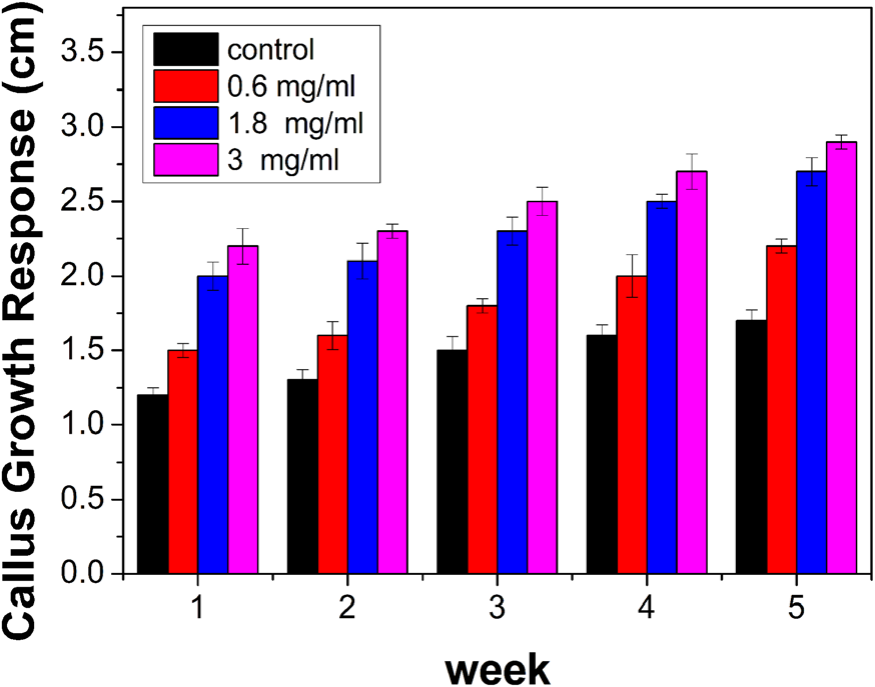
Graph illustrating the development of callus growth over successive weeks.

The results indicate that callus growth was enhanced when ACDs were added as compared to the controlled growth (Fig. 9). It also suggests that CDs have a positive effect on plant tissue culture. A dose-dependent response was also observed, with higher concentrations of ACDs (1.8 mg mL^−1^ and 3 mg mL^−1^), demonstrating a more pronounced increase in cellular proliferation compared to the lower concentration (0.6 mg mL^−1^). The unique properties of CDs such as their small size, high surface area and specific chemical composition will be stimulating cell division and growth.

## 4. Conclusions

In summary, we have facilely synthesised ACDs by microwave assisted method using tomato as the carbon source and amino acid as the dopant. We have thoroughly investigated the properties of ACDs using UV-visible optical absorption, PL, FTIR, XPS, XRD and TEM techniques. The MTT assay against L-929 normal cells verified the low cytotoxicity of the particles. The effects of ACDs on the germination rate and growth rate of pea plants were investigated. It was observed that at low concentrations, ACDs enhanced both the germination rate and growth of seedlings. The incorporation of ACD into the tissue culture medium resulted in a significant increase in callus growth, suggesting a stimulating effect of ACDs on callus proliferation. The observation not only underscores the potential utility of ACDs as a growth-promoting agent in tissue culture but also opens new avenues for research and applications in the field.

## Data availability

The data supporting this article have been included as part of the ESI.†

## Conflicts of interest

There are no conflicts to declare.

## Supplementary Material

RA-015-D4RA08776C-s001
