# Supplementary material for: Amino acid assisted synthesis of CDs: a novel paradigm in plant tissue culture media for enhanced cellular effects and biotechnological advancements
Source: RSC Adv. 2025 Jan 28;15(4):2759–65. doi: 10.1039/d4ra08776c (PMC11774188; doi:10.1039/d4ra08776c)
Supplement: RA-015-D4RA08776C-s001 [file RA-015-D4RA08776C-s001.pdf]

Supplementary Information (SI) for RSC Advances.
This journal is © The Royal Society of Chemistry 2025

Supporting Information

Amino acid assisted synthesis of carbon dots: A novel paradigm in plant tissue culture media for enhanced cellular effects and biotechnological advancements

Annmary C[a], Bitty Joseph[a,b], N.J. Simi[c], V.V Ison[a,d]

Centre for Nano Bio Polymer Science and Technology, Department of Physics, St. Thomas College, Palai, Kerala-686574, India

Department of Physics, St. George's College, Aruvithura, Kerala- 686122, India

Department of Physics, Newman College, Thodupuzha, Kerala- 685585, India

Department of Physics, Kuriakose Elias College, Mannanam, Kerala-686561, India.

E-mail: isonv@rediffmail.com; Tel: +919446126926

Micropropagation

Collection of plants

Healthy plants of Oncidium brassia were raised in pots containing soil under greenhouse conditions at Athmic Biotech Solutions. The explants taken from these plants were used for micro propagation experiments.

Pre-propagation stage

The pre propagation stage requires proper maintenance of the mother plants in the green house under disease and insect free conditions with minimal dust. Clean enclosed areas, plastic tunnels and net covered tunnels provide high quality explant source plants with minimal infection. Collection of explants for micro propagation should be done after appropriate pre-treatment of the mother plants with fungicides and pesticides to minimize contamination in the *invitro* cultures. This improves growth and multiplication rates of in vitro cultures.

Explant preparation

The explants (seeds) were washed thoroughly with water (5-7 minutes) and treated with the detergent Tween-20 for 10 minutes. Following that, the explants were rinsed thrice with distilled water to remove excess detergent. To avoid bacterial and fungal contamination, it was further treated with bavistin (100 mg/100 mL) for 15-20 minutes and with 2% sodium hypochloride solution for 7-8 minutes under continuous stirring, immersed in 0.1% (w/v) mercuric chloride ($HgCl_2$) for few minutes for surface sterilization. Finally, they were washed repeatedly with distilled water thrice to remove the disinfectants properly. The cut ends of the explants were again trimmed with the help of sterile blade to eliminate any possible residue of surfactant and the explants were used for culturing.

Test sample preparation

5 mL of the test sample was made up to 10 mL using distilled water. From these different concentrations were taken for analysis.

Explant used for inoculation: Callus developed from seeds of *Oncidium Brassia* was used for this particular study.

Tissue culture media

Following concentrations to be tried in explants of ONCIDIUM BRASSIA

- Half MS+ 2 mg/L BAP + 0.6 mg/mL Test sample +Sucrose full Strength.
- Half MS+ 2 mg/L BAP + 1.2 mg/mL Test sample +Sucrose full Strength.
- Half MS+ 2 mg/L BAP + 1.8 mg/mL Test sample +Sucrose full Strength.
- Half MS+ 2 mg/L BAP + 2.4 mg/mL Test sample +Sucrose full Strength.
- Half MS+ 2 mg/L BAP + 3 mg/mL Test sample +Sucrose full Strength.

MS nutrient stock

Nutrient salts and vitamins are prepared as stock solutions (20X or 200X concentrations of that required in the medium) as specified. The stocks are stored at 4 ºC. The desired amount of concentrated stocks is mixed to prepare 1 L of medium.

**MS major salts**	**mg/L**	**500 ml stock (20X)**
NH_4NO_3	1650 mg	16.5 gm
KNO_3	1900 mg	19 gm
$CaCl_2.2H_2O$	440 mg	4.4 gm
$MgSO_4.7H_2O$	370 mg	3.7 gm
KH_2PO_4	170 mg	1.7 gm
**MS minor salts**	**mg/L**	**500 ml stock (200X)**
H_3BO_3	6.2 mg	620 mg
$MnSO_4.4H_2O$	22.3 mg	2230 mg
$ZnSO_4.4H_2O$	8.6 mg	860 mg
KI	0.83 mg	83 mg
$Na_2MoO_4.2H_2O$	0.25 mg	25 mg
$CoCl_2.6H_2O$	0.025 mg	2.5 mg

$CuSO_4.5H_2O$	0.025 mg	2.5 mg
**MS Vitamins**	**mg/ L**	**500 mL stock (200X)**
Thiamine (HCl)	0.1 mg	10 mg
Niacine	0.5 mg	50 mg
Glycine	2.0 mg	200 mg
Pyrodoxine (HCl)	0.5 mg	50 mg

Plant growth regulators

The plant growth regulators are important in plant tissue culture since they play vital roles in stem elongation, tropism and apical dominance.

- **Auxins:** Essential for cell division ,cell elongation, cell differentiation ,organogenesis and embryogenesis, callus formation
- **Cytokinins:** Cytokinins promote cell division, shoot proliferation and influence the cell cycle.

pH of tissue culture media

pH is adjusted between 5 to 7 before gelling and sterilization with the help of dilute NaOH.

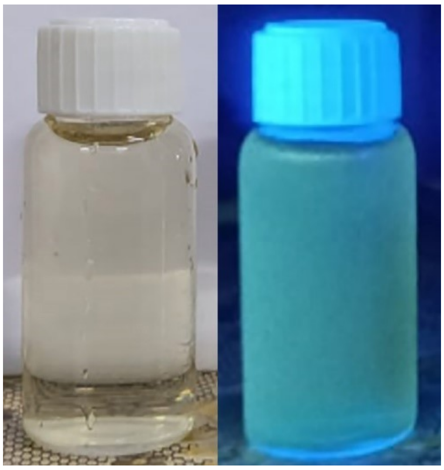

Fig.S1. ACDs under day light and UV excitation

Reference

Murashige T & Skoog F (1962) A revised medium for rapid growth and bioassays with tobacco tissue cultures. Physiol. Plant 15: 473-497.
